# Pseudogene INTS6P1 regulates its cognate gene INTS6 through competitive binding of miR-17-5p in hepatocellular carcinoma

**DOI:** 10.18632/oncotarget.3290

**Published:** 2015-01-21

**Authors:** Haoran Peng, Masaharu Ishida, Ling Li, Atsushi Saito, Atsushi Kamiya, James P. Hamilton, Rongdang Fu, Alexandru V. Olaru, Fangmei An, Irinel Popescu, Razvan Iacob, Simona Dima, Sorin T. Alexandrescu, Razvan Grigorie, Anca Nastase, Ioana Berindan-Neagoe, Ciprian Tomuleasa, Florin Graur, Florin Zaharia, Michael S. Torbenson, Esteban Mezey, Minqiang Lu, Florin M. Selaru

**Affiliations:** ^1^ Division of Gastroenterology and Hepatology, Department of Medicine, The Johns Hopkins Hospital, Baltimore, Maryland, USA; ^2^ Department of Psychiatry and Behavioral Sciences, The Johns Hopkins Hospital, Baltimore, Maryland, USA; ^3^ Liver Transplantation Center, The Third Affiliated Hospital of Sun Yat-Sen University, Guangzhou, Guangdong, P.R. China; ^4^ Department of Gastroenterology, Wuxi People's Hospital Affiliated to Nanjing Medical University, Wuxi, Jiangsu, P.R. China; ^5^ Dan Setlacec Center of General Surgery and Liver Transplantation, Fundeni Clinical Institute, Bucharest, Romania; ^6^ Department of Immunology, The Iuliu Hatieganu University of Medicine and Pharmacy, Cluj Napoca, Romania; ^7^ Department of Functional Genomics, The Oncology Institute Ion Chiricuta, Cluj Napoca, Romania; ^8^ The Research Center for Functional Genomics, Biomedicine and Translational Medicine, The Iuliu Hatieganu University of Medicine and Pharmacy, Cluj Napoca, Romania; ^9^ Department of Hematology, The Oncology Institute Ion Chiricuta, Cluj Napoca, Romania; ^10^ Department of Surgery, The Iuliu Hatieganu University of Medicine and Pharmacy, Cluj Napoca, Romania; ^11^ Department of Surgery, Regional Institute of Gastroenterology and Hepatology “Octavian Fodor”, Cluj Napoca, Romania; ^12^ Department of Pathology, The Johns Hopkins Hospital, Baltimore, Maryland, USA; ^13^ The Sidney Kimmel Comprehensive Cancer Center, The Johns Hopkins Hospital, Baltimore, Maryland, USA

**Keywords:** hepatocellular carcinoma, pseudogene, tumor suppressor, competitive endogenous RNA, INTS6

## Abstract

The complex regulation of tumor suppressive gene and its pseudogenes play key roles in the pathogenesis of hepatocellular cancer (HCC). However, the roles played by pseudogenes in the pathogenesis of HCC are still incompletely elucidated. This study identifies the putative tumor suppressor INTS6 and its pseudogene INTS6P1 in HCC through the whole genome microarray expression. Furthermore, the functional studies – include growth curves, cell death, migration assays and *in vivo* studies – verify the tumor suppressive roles of INTS6 and INTS6P1 in HCC. Finally, the mechanistic experiments indicate that INTS6 and INTS6P1 are reciprocally regulated through competition for oncomiR-17-5p. Taken together, these findings demonstrate INTS6P1 and INTS6 exert the tumor suppressive roles through competing for oncomiR-17-5p. Our investigation of this regulatory circuit reveals novel insights into the underlying mechanisms of hepatocarcinogenesis.

## INTRODUCTION

In the year 2013, it is estimated that there were 30,640 new diagnoses of primary liver cancer and 21,670 people will die as a consequence of this cancer in the United States (http://www.cancer.gov/). Hepatocellular carcinoma (HCC) accounts for the vast majority of primary liver cancer cases. Tremendous progress has been made recently towards understanding the basic mechanisms of HCC genesis and homeostasis [1, 2]. Sequencing efforts have been crucial in revealing a plethora of mutated genes in HCC, such as ARID2, ARID1A, CTNNB1, and others [3-5]. The study of gene expression in HCC has proven equally fruitful, with several new types of HCC based on mRNA levels being defined [6]. Lastly, epigenetic changes are identified in HCC and have an ascribed etiologic role [7]. Long non-coding RNAs (lncRNAs) are a relatively recently discovered class of non-protein coding transcripts that contain more than 200 nucleotides [8]. Although lncRNAs have recently attracted a substantial amount of attention, further studies are needed to elucidate their etiologic involvement in cancer [9-12].

Studies of non coding RNA species, including microRNAs (miRs) and lncRNAs, have ushered in an era of sophistication in our understanding of a complex and previously underappreciated level of protein regulation. MiRs are known to down-regulate coding transcripts and therefore play major regulatory roles in health and disease [7]. The realization of certain long non coding transcripts can function as “sponges” for certain miR species, suggested that these non coding RNA species might indirectly, yet effectively, regulate protein expression. According to this newly recognized competitive endogenous RNA (ceRNA) paradigm, long non-coding transcripts share miR responsive elements (MREs) with protein coding genes, and compete with these protein-coding genes for the pool of shared miRs [13]. Thus, ceRNA species bear the potential of playing crucial roles in the regulation of their cognate genes [13-16].

Pseudogenes represent an intriguing class of lncRNAs. They are structurally similar to their parental protein coding genes, with the important difference that they do not have a protein coding domain [17]. Therefore, they have been historically labeled as nonfunctional transcriptional relics [18]. The ceRNA paradigm, however, has refocused the attention on pseudogenes, since their striking sequence homology with the cognate genes renders them prime candidates for mRNA regulation through competition for shared miR species [13]. Several reports have described salient roles of pseudogenes in cancer biology [16, 19].

In the current study, we utilized an unbiased approach to identify lncRNA pseudogenes and study their putative ceRNA function in HCC. Herein, we report a novel regulatory network in HCC, comprised of integrator complex subunit 6 (INTS6), integrator complex subunit 6 pseudogene 1 (INTS6P1), and a shared miR species, miR-17-5p.

## RESULTS

### INTS6, INTS6P1 and miR-17-5p display coordinated expression in human HCC tissues

**Figure 1:**
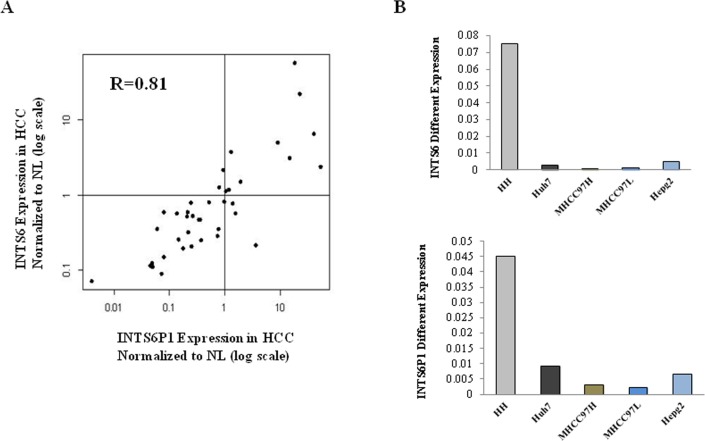
INTS6 and INTS6P1 are putative tumor suppressors in HCC (A) The expression levels of INTS6 and INTS6P1 are assayed in 39 pairs of HCC and paired normal liver tissue (NL). X-Axis – INTS6P1 expression levels in HCC tissues normalized to paired NL tissues. Y-Axis – INTS6 expression levels in HCC tissues normalized to paired NL tissues. Both X- and Y-axis are in log units. INTS6 and INTS6P1 are expressed at lower levels in approximately 70% HCC *vs.* NL samples (in the figure, the dots representing specimens located in the left lower quadrant). Moreover, the expression of INTS6 is positively correlated with INTS6P1 in HCC. The R value (correlation coefficient value) is 0.81 (Spearman correlation). Data presented is normalized to respective gene expression in corresponding NL tissues. (B) The expression level of INTS6 and INTS6P1 is lower in HCC cells (Huh7, MHCC97H, MHCC97L, and HepG2) compared to normal human hepatocytes (HH).

Messenger RNA, miR and lncRNA arrays were performed on 3 pairs of HCC and matched normal liver tissues (the data have been deposit to GEO: GSE64633). INTS6 and INTS6P1 were found to be down-regulated in HCC *vs.* matched normal tissues (Supporting document 1: Table 1). In addition, miR-17-5p was found to be up-regulated in same HCC *vs.* matched normal liver tissues (Supporting document 1: Table 1). To further scrutinize potential mechanistic explanations for the coordinated expression levels of INTS6, INTS6P1 and miR-17-5p, we investigated the nucleotide sequence of INTS6 and INTS6P1. As predicted by the Genome browser (UCSC) and NCBI Blast, INTS6P1 displays 96% homology with the ORF of INTS6 (Supporting document: Figure 1). Of further importance, miRcode predicts a miR-17-5p binding site in INTS6P1 as well as in the open reading frame (ORF) of INTS6. Therefore, we hypothesized that INTS6P1 might regulate the expression of INTS6, through competing for the available quantity of miR-17-5p.

### INTS6P1 positively correlates with INTS6 in a large cohort of human HCC tissues

To validate the array data, the expression of INTS6 and INTS6P1 was assayed with qRT-PCR in 39 pairs of human HCC and matched normal liver tissues. The expression of both INTS6P1 and INTS6 was not only down-regulated in approximately 70% of HCC *vs.* normal liver tissues, but there was also positive correlation between the expression of both gene and the pseudogene (R=0.81, Figure 1A). Moreover, the expression of INTS6 as well as INTS6P1 was down-regulated in HCC cell lines (Huh7, MHCC97H, MHCC97L, and HepG2) when compared to normal human hepatocytes (HH) (Figure 1B). The positive correlation between expression of INTS6 and INTS6P1, suggests that these 2 genes may be part of a regulatory circuit.

### INTS6 as well as INTS6P1 exert tumor suppressive effects on HCC cells *in vitro*

**Figure 2:**
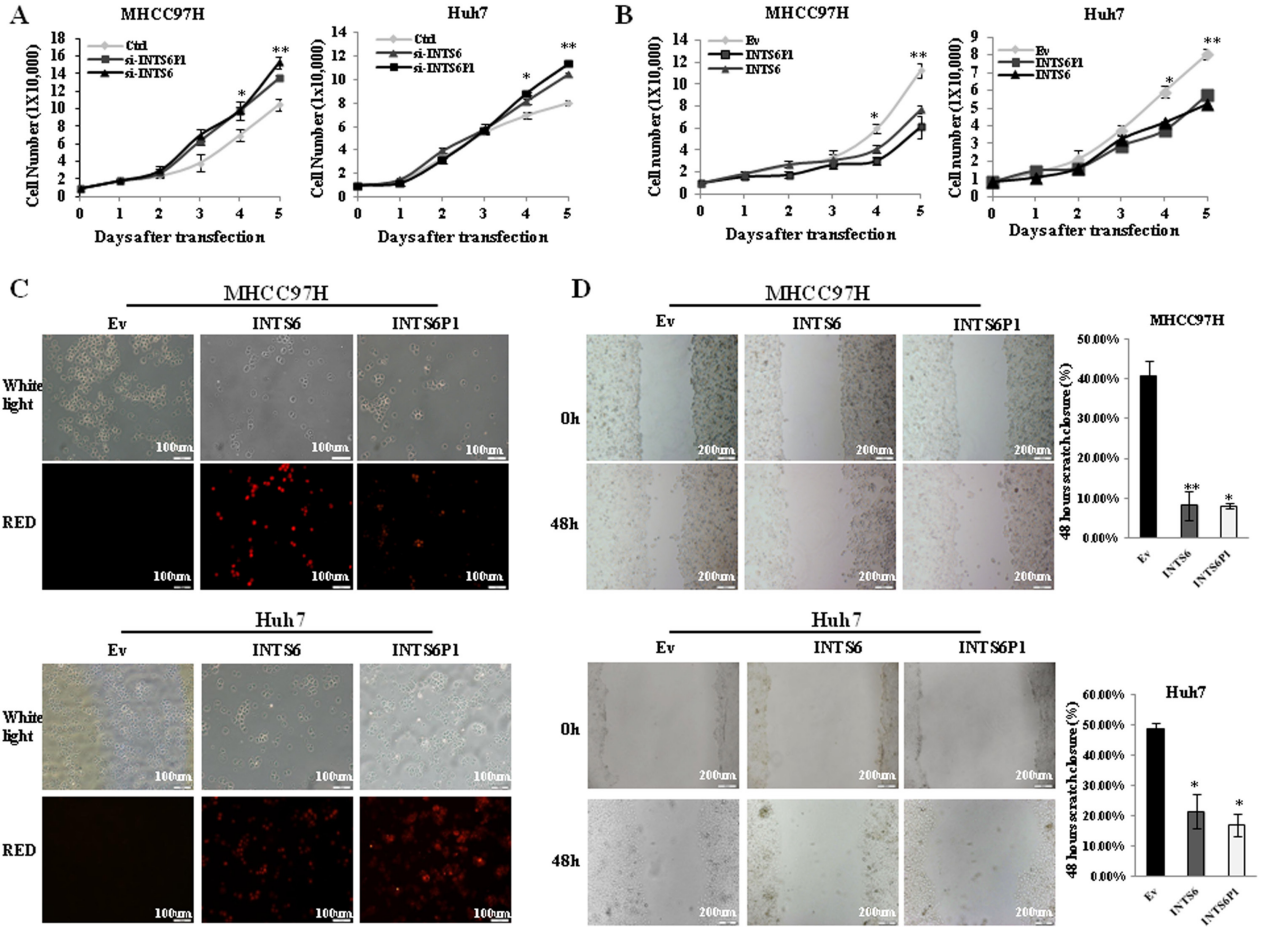
INTS6, as well as INTS6P1, suppress the growth and mobility of HCC cells (A) Loss of function of INTS6 (triangle in the figure) or INTS6P1 (square in the figure) induced by specific siRNA species results in increased cell growth. At day 4, there was a statistically significant difference in number of MHCC97H cells between Ctrl (control) cells and cells transfected with either si-INTS6 (p = 0.37) or si-INTS6P1 (p = 0.45) (*left*), as well as in Huh7 cells transfected with si-INTS6 (p = 0.027) or si-INTS6P1 (p = 0.012) (*right*). At day 5, there was a statistically significant difference in number of MHCC97H cells between Ctrl (control) cells and cells transfected with either si-INTS6 (p = 0.0039) or si-INTS6P1 (p = 0.0018) (*left*), as well as in Huh7 cells transfected with si-INTS6 (p = 0.0026) or si-INTS6P1 (p = 0.0014) (*right*). Error bars represent mean ± SD. (B) Up-regulation of INTS6 or INTS6P1 suppresses the growth of MHCC97H at day 4 (p = 0.016 with INTS6, p = 0.015 with INTS6P1) (*left*), as well as Huh7 when compared to the negative control (Ev) (p = 0.0089 with INTS6, p = 0.0045 with INTS6P1) (*right*). At day 5, the growth of MHCC97H is suppressed dramatically by INTS6 (p = 0.0015) or INTS6P1 (p = 0.003) (*left*), the growth of Huh7 is suppressed by INTS6 (p = 0.0024) or INTS6 (p = 0.0076) as well (*right*) Error bars represent mean ± SD. (C) Up-regulation of INTS6 or INTS6P1 increases cell death when compared to the negative control (Ev). (D) The migration of MHCC97H cells is significantly suppressed by over-expressing INTS6 (p = 0.018) or INTS6P1 (p = 0.0041) (*top*). The migration of Huh7 cells was significantly suppressed by over-expressing INTS6 (p = 0.01) or INTS6P1 (p = 0.026) (*bottom*). The wound closure distance is quantified by ImageJ. Error bars represent mean ± SD.

Previous studies suggested the tumor suppressive role for INTS6 in other cancer types [20-23], however, to date, there are no studies to report its role in HCC. Depletion of INTS6 *via* siRNA significantly increased cell growth in MHCC97H as well as in Huh7 cells. Moreover, siRNA-mediated down-regulation of INTS6P1 similarly increased cell growth in MHCC97H and Huh7 cells (Figure 2A, Supporting document 1: Figure 2A and B). In gain of function studies performed in the same 2 cell lines, the up-regulation of INTS6, as well as the up-regulation of INTS6P1, respectively, induced growth arrest (Figure 2B). In a different set of experiments, HCC cells were transfected with INTS6P1 or INTS6 and investigated for cell death. We noted that over-expression of either INTS6 or INTS6P1 induced an increase in cell death when compared to the negative control (Figure 2C). Finally, to study the effect of INTS6P1 or INTS6 on the mobility of HCC cells, we conducted scratch assays on HCC cells transfected with INTS6P1 or INTS6. In comparison with the negative control, HCC cells with either INTS6P1 or INTS6 over-expression, respectively, migrated less (Figure 2D). Taken together, these findings suggest that INTS6P1 and INTS6 exert tumor suppressive effects by promoting HCC cell death and inhibiting cell mobility.

### INTS6 as well as INTS6P1 exert tumor suppressive effects on HCC cells *in vivo*

**Figure 3:**
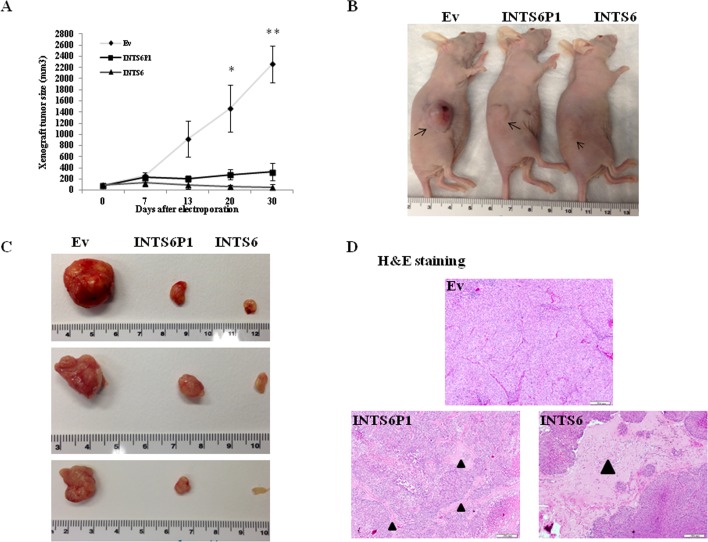
INTS6P1 and INTS6 exert tumor suppressive activity *in vivo* (A) Tumors established in nude mice grow significantly less when INTS6 or INTS6P1 are up-regulated *in vivo.* At day 20, there was a statistically significant difference in tumor size between Ev (control) and tumors electroporated with either INTS6 plasmid (p = 0.023) or INTS6P1 plasmid (p = 0.037). At day 30, there was a statistically significant difference in size of tumors between Ev (control) group and tumors electroporated with either INTS6 plasmid (0.006) or INTS6P1 plasmid (p = 0.0038). Error bars represent mean ± SD. (B, C) Representative images demonstrate the size of control tumors (Ev) compared to tumors treated *in vivo* with INTS6 or INTS6P1. (D) The H&E staining of tumors show lower cross sectional cancer component in tumors treated with INTS6 or INTS6P1 *in vivo.* The black triangles indicate a higher percent of tumor stroma (and lack of cancer cells) in tumors treated with INTS6 or INTS6P1.

Electroporation has been demonstrated as one of the most efficient approaches in delivering plasmid DNA *in vivo* [24]. After allowing xenograft tumors to grow in nude mice, we employed electroporation to up-regulate the expression of INTS6 and INTS6P1, respectively. Since day 20, the growth of tumors in which INTS6 was up-regulated was significantly less *vs.* control tumors (p<0.05). In addition, up-regulation of INTS6P1 *in vivo* induced a similar, albeit of smaller magnitude, decrease in growth (Figure 3A-C). Furthermore, tumors in which INTS6 or INTS6P1 was up-regulated, displayed a lower cross sectional cancer component, when compared to control tumors (Figure 3D).

### The expression levels of INTS6P1 and INTS6 are functionally correlated in HCC cells

**Figure 4:**
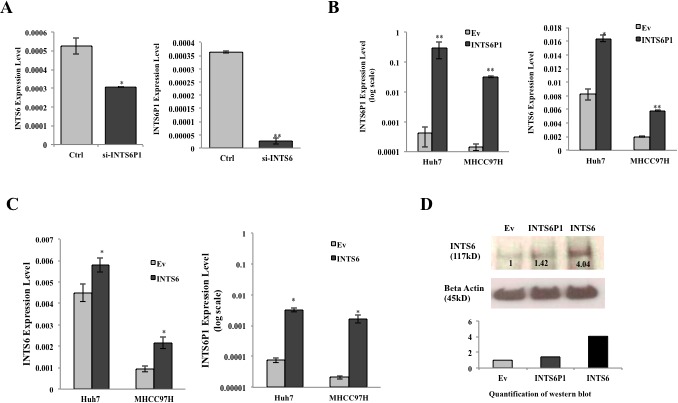
INTS6 and INTS6P1 are a part of the regulatory circuit (A) The inhibition of INTS6P1 by si-RNA suppresses the expression of INTS6 (p = 0.0089) (*left*). Down-regulation of INTS6 suppresses the expression of INTS6P1 (p = 0.02) (*right*). Error bars represent mean ± SD. (B) The over-expression of INTS6P1 by transfected INTS6P1 (*MIEG3-INTS6P1*) in HCC cells (left) enhances the expression level of INTS6 compared to negative control cells (*MIEG3-Ev*) (p = 0.025 in Huh7, p = 0.0017 in MHCC97H) (*right*). Error bars represent mean ± SD. (C) The over-expression of INTS6 by transfected INTS6 (*MIEG3-INTS61*) in HCC cells (*left*) enhances the expression level of INTS6P1 compared to negative control cells (*MIEG3-Ev*) (p = 0.013 in Huh7, p = 0.031 in MHCC97H) (*right*). Error bars represent mean ± SD. (D) The over-expression of INTS6P1 increases the transcriptional expression of INTS6, as well as the the protein level of INTS6. Western blot is quantified by ImageJ.

To investigate if INTS6 and INTS6P1 are part of the same regulatory circuit, we modulated the expression levels of either INTS6 or INTS6P1, and recorded the effects onto the expression levels of INTS6P1 or INTS6, respectively. The inhibition of INTS6 induced significant suppression of INTS6P1, by contrast, the down-regulation of INTS6P1 induced significant suppression of INTS6 (Figure 4A). To further investigate the co-regulation of INTS6 and INTS6P1, in gain of function studies, we noted that the up-regulation of INTS6P1 significantly increased the expression level of INTS6 (Figure 4B). Lastly, the over-expression of INTS6 induced up-regulation of INTS6P1 (Figure 4C). The over-expression of INTS6P1 did not only increase the transcriptional level of INTS6, but also enhanced the protein level of INTS6 (Figure 4D). Taken together, these findings suggest that the expression of INTS6P1 and INTS6 are functionally co-regulated by a yet to be determined mechanism.

### INTS6P1 regulates the expression of INTS6 through competition for miR-17-5p

**Figure 5:**
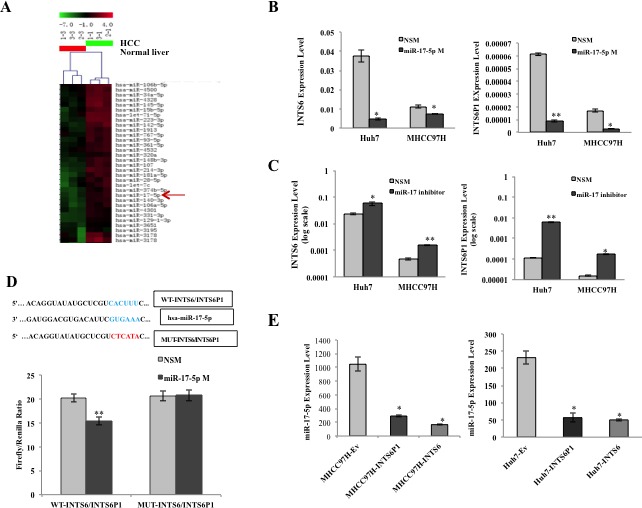
MiR-17-5p mediates the regulation of INTS6 and INTS6P1 (A) Heat map representation of microarray data demonstrates that miR-17-5p is up-regulated in HCC *vs.* normal liver tissues. (B) Up-regulation of miR-17-5p results in the suppression of INTS6 (p = 0.049 in Huh7, p = 0.023 in MHCC97H) (*left*), as well as INTS6P1 in HCC cells (p = 0.00017 in Huh7, p = 0.04 in MHCC97H) (*right*). Error bars represent mean ± SD. (C) The depletion of miR-17-5p de-represses the expression of INTS6 (p = 0.021 in Huh7, p = 0.0055 in MHCC97H) (*left*), as well as INTS6P1 in HCC cells (p = 0.009 in Huh7, p = 0.023 in MHCC97H) (*right*). Error bars represent mean ± SD. (D) (*Top*) miR-17-5p seed sequence (blue in the figure) is shown, along with the binding site in INTS6 and INTS6P1 (WT-INTS6/INTS6P1, blue), and the mutated miR-17-5p binding (MUT-INTS6/INTS6P1, red). (*Bottom*) miR-17-5p binds to the wild type binding site in INTS6/INTS6P1 as shown by a decrease of luciferase activity *vs.* treatment with non specific mimic (NSM, left side of the graph). However, upon mutating the binding site in INTS6 or INTS6P1, miR-17-5p fails to bind to these constructs (right side of the graph) (p = 0.0093). Error bars represent mean ± SD. (E) The expression level of miR-17-5p is strongly inhibited by up-regulating INTS6 (p = 0.029) or INTS6P1 (p = 0.031) in MHCC97H cells (*left*). The expression level of miR-17-5p is inhibited by up-regulated INTS6 (p = 0.039) or INTS6P1 (p = 0.016) in Huh7 cells as well (*right*). Error bars represent mean ± SD.

The array data suggested that, in human HCC specimens, INTS6 and INTS6P1 are down-regulated, while miR-17-5p is up-regulated, when compared to the matched normal liver tissues (Figure 5A). Since expression level changes in either INTS6 or INTS6P1 induce a reciprocal expression change in the counterpart gene, we hypothesized that miR-17-5p, which has a binding site in INTS6, as well as INTS6P1, could be the regulatory factor. In accord with this hypothesis, we noted that increasing the expression of miR-17-5p induced down-regulation of INTS6, as well as INTS6P1 (Figure 5B, Supporting document 1: Figure 2C). Furthermore, inhibition of miR-17-5p induced de-repression and subsequent up-regulation of both INTS6 and INTS6P1 (Figure 5C, Supporting document 1: Figure 2D). Next, we asked if miR-17-5p affects INTS6 and INTS6P1 directly or indirectly. To this end, we cloned a fragment of the homologous sequence of INTS6P1 and INTS6, which contained the miR-17-5p binding site, into a luciferase vector. We noted that treatment with miR-17-5p down-regulated the luciferase activity of both INTS6 as well as INTS6P1. However, mutating the binding site of miR-17-5p rescued the luciferase activity, strongly supporting a direct interaction between miR-17-5p and this binding site in INTS6 as well as in INTS6P1 (Figure 5D, and Supporting document 1: Figure 3). Furthermore, the up-regulation of either INTS6 or INTS6P1 resulted in a marked decrease of miR-17-5p, in accord with the hypothesis that either of these genes can act as a sponge for the available cellular miR-17-5p transcripts (Figure 5E).

## DISCUSSION

LncRNA species represent a relatively newly discovered type of non coding RNA transcript [12]. Several studies reported functional roles for lncRNAs in human cancer [8, 25]. In HCC, however, to date, only a few studies implicated lncRNAs, such as MALAT1, HOTAIR, HOTTIP/HOXA13, H19, HULC, MEG3, in the cancer pathogenesis [9, 11, 26-29]. In part, this is due to challenges characteristic to the lncRNA field. Studies to date demonstrated a wide variety of mechanisms of action. However, the lack of a unifying theme precludes a systematic search for lncRNA mechanisms of action [8]. The newly emerged ceRNA paradigm suggests that lncRNAs with high sequence complementarity to protein coding genes, are prime candidates to be investigated as ceRNA [13]. The current study represents the first systematic effort to identify functional networks composed of triplet species: miRs, lncRNAs and mRNAs. The unbiased approach presented herein represents a novel methodology in unraveling the function of lncRNAs in HCC.

Pseudogenes are non coding RNA transcripts that have historically been considered to be nonfunctional [18]. However, similar to other non coding RNA species, such as miRs, pseudogenes are now known to play important regulatory roles [30]. Since the discovery of the first pseudogene with regulatory functions, significant efforts have been directed at elucidating the effect of pseudogenes in the regulation of protein expression [15, 16, 30]. Pseudogene transcripts, as members of the ceRNA network, participate in the regulation of their parental coding genes through competitive binding to shared miRs. In other words, the over-expression of pseudogene transcripts increases the abundance of MREs and results in de-repression of their parental protein coding genes.

Several studies have suggested that INTS6 plays tumor suppressive roles in several human cancers, such as non-small cell lung carcinoma, esophageal squamous cell carcinoma and prostate carcinoma [20-23, 31-34]. Mechanistically, INTS6 appears to induce Gap 1 (G1) arrest, explaining, in part its tumor suppressive roles in prostate cancer [20]. Furthermore, the low expression level of INTS6 in prostate cancer has been found to be caused, in part, by promoter region CpG hypermethylation [34]. However, the role of INTS6 in HCC is still largely not understood. Here, we report that INTS6 plays a critical role as a tumor suppressor in HCC. In addition, this study brings mechanistic evidence showing that INTS6 inhibits HCC cell growth, migration and survival. Since a majority of human HCC tissues analyzed in this study demonstrate down-regulation of INTS6, we conclude that INTS6 likely plays a major role in HCC, warranting further studies.

INTS6P1 has never been reported as a tumor suppressive non coding gene in any human cancer. The current study, for the first time, strongly argues that INTS6P1 plays a tumor suppressive role in HCC by suppressing HCC cell growth, migration and survival. The data presented herein warrants further studies in other human cancers, in particular its cognate gene, INTS6, is found to be a tumor suppressor. Of note, in strong support of our assertion that INTS6 and INTS6P1 are part of a regulatory circuit, their expression is highly correlated in the human HCC and matched normal liver specimens. Finally, we present evidence to show that INTS6P1 regulates the tumor suppressor INTS6 by competitive binding of miR-17-5p, a known oncomiR in HCC [35-38].

## CONCLUSIONS

The current study reports, for the first time, that INTS6 and its pseudogene INTS6P1 are tumor suppressors in HCC. In addition, we bring evidence to show that INTS6P1 post-transcriptionally regulates the expression level of INTS6 through competitive binding to the oncogenic miR-17-5p in HCC. These findings provide new insights into complex regulatory networks in HCC and uncover novel therapeutic targets.

## MATERIALS AND METHODS

### Human Tissues

After informed consent, and under the Institutional Review Board approval, human HCC and paired normal liver tissues were obtained immediately after surgery at the Johns Hopkins Hospital (Baltimore, US), The 3^rd^ Affiliated Hospital of Sun Yat-sen University (Guangzhou, China), Fundeni Clinical Institute (Bucharest, Romania), and Ion Chiricuta Comprehensive Cancer Center (Clui Nanoca, Romania). Tissues were snap frozen upon acquisition and stored in a −80°C freezer till use.

### Cells and Culture Conditions

The hepatoma cell lines, MHCC97H (a gift from Dr. Yoke Peng Loh, NIH, and Liver Cancer Institute, Fudan University) and Huh7 were grown in Dulbecco's Modified Eagle's Medium (DMEM, Corning Cellgo) supplemented with 10% fetal calf serum FCS, 1000 U/ml penicillin/streptomycin (P/S, Quality Biological). All cell lines were cultured at 37°C in a humidified atmosphere with 5% CO_2_.

### LncRNA, mRNA labeling and Array Hybridization

The Arraystar Human lncRNA Array v2.0 was used to profile both lncRNAs and messenger RNAs (mRNAs) in human genome of 3 pairs of human HCC and the matched normal tissues. 33,045 LncRNAs were collected from the authoritative data sources including RefSeq, UCSC knowngenes, Ensembl and many related literatures. Sample labeling and array hybridization were performed according to the Agilent One-Color Microarray-Based Gene Expression Analysis protocol (Agilent Technology) with minor modifications. Briefly, mRNA was purified from 1 μg total RNA after removal of rRNA (mRNA-ONLY™ Eukaryotic mRNA Isolation Kit, Epicentre). Then, each sample was amplified and transcribed into fluorescent cRNA along the entire length of the transcripts without 3′ bias utilizing a random priming method. The labeled cRNAs were purified by RNAeasy Mini Kit (Qiagen). The concentration and specific activity of the labeled cRNAs (pmol Cy3/μg cRNA) were measured by NanoDrop ND-1000. 1 μg of each labeled cRNA is fragmented by adding 11 μl 10 × Blocking Agent and 2.2 μl of 25 × Fragmentation Buffer, then heated the mixture at 60°C for 30 min, finally 55 μl 2 × GE Hybridization buffer is added to dilute the labeled cRNA. 100 μl of hybridization solution was dispensed into the gasket slide and assembled to the lncRNA expression microarray slide. The slides were incubated for 17 hours at 65°C in an Agilent Hybridization Oven. The hybridized arrays were washed, fixed and scanned with using the Agilent DNA Microarray Scanner (part number G2505B).

### MicroRNA Labeling and Array Hybridization

MiRCURY™ Hy3™/Hy5™ Power labeling kit (Exiqon) was used according to the manufacturer's guideline for miR labelling of the same 3 pairs of HCC and matched normal liver tissues. 1.0 mg of each sample was 3′-end-labeled with Hy3^TM^ fluorescent label, using T4 RNA ligase by the following procedure: RNA in 2.0 μL of water was combined with 1.0 μl of CIP buffer and CIP (Exiqon). The mixture was incubated for 30 min at 37°C, and was terminated by incubation for 5 min at 95°C. Then 3.0 μL of labeling buffer, 1.5 μL of fluorescent label (Hy3^TM^), 2.0 μL of DMSO, 2.0 μL of labeling enzyme were added into the mixture. The labeling reaction was incubated for 1 hour at 16°C, and terminated by incubation for 15 min at 65°C. After stopping the labeling procedure, the Hy3^TM^-labeled samples were hybridized on the miRCURY^TM^ LNA Array (v.18.0) (Exiqon) according to array manual. The total 25 μL mixture from Hy3^TM^-labeled samples with 25 μL hybridization buffer were first denatured for 2 min at 95°C, incubated on ice for 2 min and then hybridized to the microarray for 16 – 20 hours at 56°C in a 12-Bay Hybridization Systems (Hybridization System - Nimblegen Systems), which provides an active mixing action and constant incubation temperature to improve hybridization uniformity and enhance signal. Following hybridization, the slides were achieved, washed several times using Wash buffer kit (Exiqon), and finally dried by centrifugation for 5 min at 400 rpm. Then the slides were scanned using the Axon GenePix 4000B microarray scanner (Axon Instruments).

### Transient Transfection

To transfect the HCC cells with siRNA or siRNA control, miR mimic, non specific mimic (NSM) or miR inhibitor (Thermo Scientific), Huh7 or MHCC97H cells were seeded onto 12-well plate. When the cells were getting 60~80% confluent, 100 nM siRNA or siRNA control, miR mimic, miR inhibitor or NSM were applied to the cells by combining lipofectamine iMAX (Invitrogen) according to the manufacture`s recommendations. For plasmid transfection, Huh7 or MHCC97H (3×10^5^) were seeded onto 6-well plate, the next day cells were transfected with lipofectamine 2000 (Invitrogen) according to the manufacture`s recommendations. RNA and protein were harvested in 72 hours after transfection.

### Quantitative Real-time PCR

To evaluate the expression level of INTS6, INTS6P1 and miR-17-5p, quantitative real-time PCR (qRT-PCR) was performed. The specific primer sets for INTS6 and INTS6P1 were designed from the regions that share low homology between INTS6 and INTS6P1 (Supporting document 1: Table 2). The specificity of the primers was verified by PCR the relevant genes and sequencing the PCR products (Supporting document 2). TaqMan miR Assay kits (Applied Biosystems) were used for miR-17-5p (Applied biosystems) and normalized to RNU6B (Applied biosystems). Power SYBR Green PCR Master Mix (Applied Biosystems) was used for INTS6P1 and INTS6 qRT-PCR. The expression of INTS6P1 and INTS6 was normalized to GAPDH. Relative expression of target RNAs was calculated using the delta Ct method. All PCR reactions were carried out on the 7900 HT Fast Real-time PCR System (Applied Biosystems) in duplicate.

### Western Blot

Cells were lysed in Laemmli sample buffer (BioRad) supplemented with a protease inhibitor (Roche). Protein concentration was measured using a BCA Protein Assay kit (Thermo Scientific). Cell lysates (40–45 mg per lane) were electrophoresed on 10–20% polyacrylamide gels (Bio-Rad) and transferred to Immobilon-PSQ membranes (Millipore). The membranes were blocked with TBS containing 5% skim milk and 0.1% Tween-20 (TBST), then incubated with the primary antibody anti-INTS6 (Abcam). The membranes were incubated after TBST washing with HRP-conjugated anti-mouse secondary antibody (Cellsignaling) and analyzed using enhanced chemiluminescent HRP Antibody Detect Reagent (Denvillle Scientific). The online available software ImageJ was used to quantify the density of the bands. The expression of INTS6 and INTS6P1 was normalized to that of beta-Actin (Abcam).

### Plasmid DNA Construction

MSCV-based bicistronic retroviral vector, MIEG3 was used to express INTS6P1 and INTS6 as described previously [39]. The genomic DNA was extracted from Huh7 cells and treated with RNase A (Thermo Scientific). For amplifying the specific INTS6P1 sequence, nested PCR was performed on the genomic DNA. For the nested PCR, the 1^st^ set of primers was designed from the flank intron, the 2^nd^ set of primers was designed from the INTS6P1 sequence flanked by EcoRI (5′) and XhoI (3′) (Supporting document 1: Table 2). Next, the unique amplification of INTS6 was obtained from the complementary DNA (cDNA) using PCR primers flanked by EcoRI (5′) and XhoI (3′) (Supporting document 1: Table 2). The amplicons of INTS6P1 and INTS6 were cloned into the EcoRI and XhoI multiple cloning site of MIEG3 respectively. The expression of INTS6P1 or INTS6 was linked with expression of enhanced green fluorescence protein (eGFP) via internal ribosome entry site 2 (IRES2). All plasmid DNAs were verified by doing DNA sequencing.

### PGL4.13 Luciferase Plasmid Construction

MiRcode predicted miR-17-5p has binding sites in INTS6P1 and the open reading frame (ORF) of INTS6. The fragment containing miR17 predicted binding site in INTS6P1 and INTS6 ORF (wild type) which is highly homologous was amplified by using linker primers containing XbaI restriction sites (Supporting document 1: Table 2). Amplicon was cut by XbaI and cloned into an XbaI site downstream of the firefly luciferase structural gene in vector pGL4.13 (Promega). For the mutant, the miR-17 binding site was mutated following by 2 steps PCR mutagenesis as prescribed previously [40]. All plasmid DNAs (wild-type and mutant) were verified by sequencing.

### Luciferase Reporter Assay

Cells were seeded in 8,000/well onto 96-well plates 24 hours prior to transfection. Cells were transfected with miR-17-5p mimic or NSM. 24 hours after transfection, cells were co-transfected with constructed wild type or mutated pGL4.13 vector (firefly luciferase) company with internal control pRL-CMV (Renilla luciferase, Promega) vector. 48 hours after plasmid vector transfection, the luciferase reporter assay was performed using a Dual-Luciferase Reporter Assay System (Promega). After 48 hours, luminescence activity was measured by Veritas Microplate Luminometer (Turner Biosystems), and the luminescence activity of firefly luciferase is normalized to that of Renilla luciferase.

### Cell Growth Assay

Cell growth assays were performed on Huh7 or MHCC97H transfected with plasmid DNA or siRNA or siRNA control. Cells were plated onto 24-well plates by transfecting plasmid DNA or siRNA or siRNA control. 6 hours later, cells were transferred in triplicates onto 96-well plates at a final density of 10,000 cells/well (day 0), counted daily for a total of 5 consecutive days (days 1-5) using a hemocytometer and an inverted-light microscope.

### Scratch Assay

Scratch assay was conducted on the Huh7 or MHCC97H transfected plasmid DNA. Cells were plated onto 24 well plates after transfected with plasmid DNA. On the next day (day 0), when cells reached 100% confluent, a straight line in the middle of each wells were draw using sterile 200 μl tips. Cells were maintained in completed media and maintain at 37°C in a humidified atmosphere with 5% CO_2_. The wound healing was analyzed under microscope on 24 hours and 48 hours post scratch. The online available software ImageJ was used to quantify the percentage of wound closure.

### TUNEL Assay

TUNEL assay was performed using an In Situ Cell Death Detection Kit TMR Red (Roche). Cells were processed according to the manufacturer. Briefly, 1×10^5^ cells were cytospined to the slides. Cells were fixed with 4% Paraformaldehyde, then, permeated with 0.1% Triton X-100 in 0.1% sodium citrate, then incubated at 37°C with TUNEL mixture containing TMR-dUTP. Finally, the cells were analyzed by fluorescence microscopy using an excitation wavelength in the range of 526-560 nm.

### Subcutaneous Tumor Formation

MHCC97H cells were grown in 150mm dishes. 1×10^7^ cells/mouse were re-suspended in 100 μl PBS+100 μl Matrilgel (BD). Cells were injected to the right flank of 6 week old female nude mice (Harlan) to establish subcutaneous tumors. The mice were maintained in animal facility according to Johns Hopkins University Animal Care and Use Committee. Once the tumor size reached about 80 mm^3^, the mice were divided into 3 groups randomly, labeling EV (*MIEG3*), INTS6 (*MIEG3-INTS6*) and INTS6P1 (*MIEG3-INTS6P1*) [41]. The mice were maintained in animal facility according to The Johns Hopkins University Animal Care and Use Committee.

### Intratumoral INTS6P1 and INTS6 Plasmid DNA Delivery

Intratumoral plasmid DNA delivery was performed as described previously [41]. Briefly, mice were anesthetized with 2% isoflurane. Established subcutaneous HCC was injected with 100 μg of plasmid DNA (*MIEG3* or *MIEG3-INTS6P1* or *MIEG3-INTS6*) at 1.0 μg/μl in normal saline using an insulin syringe with a 28-gauge needle. Electric pulse was delivered using an electric pulse generator (CUY21- EDIT, BEX). The parameter was set as follow: Volt: 100, P on: 050.0, P off: 950, No: 10. The shape of the pulse was a square wave, the voltage remain constant for the duration of the pulse. A pair of forceps like electrode (CUY650P3) was used to clamp the tumor on both sides. Tumor size was measured using Vernier Callipers (length and width). Tumor volume was calculated according to the equation: *L* × *W*^2^/2 (mm^3^), where *L* = length and *W* = width. The mice were sacrificed in 30 days when the tumor sizes were significant different. The tumors were extracted from the body and fix with 10% formalin. The fixed tumors were sent to Pathology lab to make the slides and follow with the H&E (hematoxyling and eosin) staining.

### Bioinformatics

The Ensembl (http://useast.ensembl.org/index.html) was used to annotate the Gene ID: ENST00000504674 to INTS6P1 and obtained its cognate gene INTS6. The University of California Santa Cruz Genome Bioinformatics Genome Browser database (www.genome.ucsc.edu) was used to search the whole sequence of INTS6P1 and INTS6. NCBI Blast (http://blast.ncbi.nlm.nih.gov/Blast.cgi) was used to analyze the homology between INTS6P1 and the ORF of INTS6. MiRcode (http://www.miRcode.org/) was used to obtain the miRs binding sites in INTS6P1 and the ORF of INTS6.

### Statistics

The significance of coefficient correlation between INTS6P1 and INTS6 expression values was calculated via a test statistic based on Spearmen correlation coefficient. Two-tailed Student's *t*-test was used to evaluate the statistical significance of differences between two groups of data in luciferase assay, qRT-PCR, scratch assay, and cell growth assay. The mean ± SD of three or more independent experiments was reported. Differences were considered significant when *P* < 0.05 (*), *P* < 0.01 (**).

## SUPPLEMENTARY FIGURES AND TABLES


