# Supplementary material for: Pseudogene INTS6P1 regulates its cognate gene INTS6 through competitive binding of miR-17-5p in hepatocellular carcinoma
Source: Oncotarget. 2015 Jan 21;6(8):5666–77. doi: 10.18632/oncotarget.3290 (PMC4467393; doi:10.18632/oncotarget.3290)
Supplement: Supplementary file 1 [file oncotarget-06-5666-s001.pdf]

Pseudogene INTS6P1 regulates its cognate gene INTS6 through competitive binding of miR-17-5p in hepatocellular carcinoma

Supplementary Material

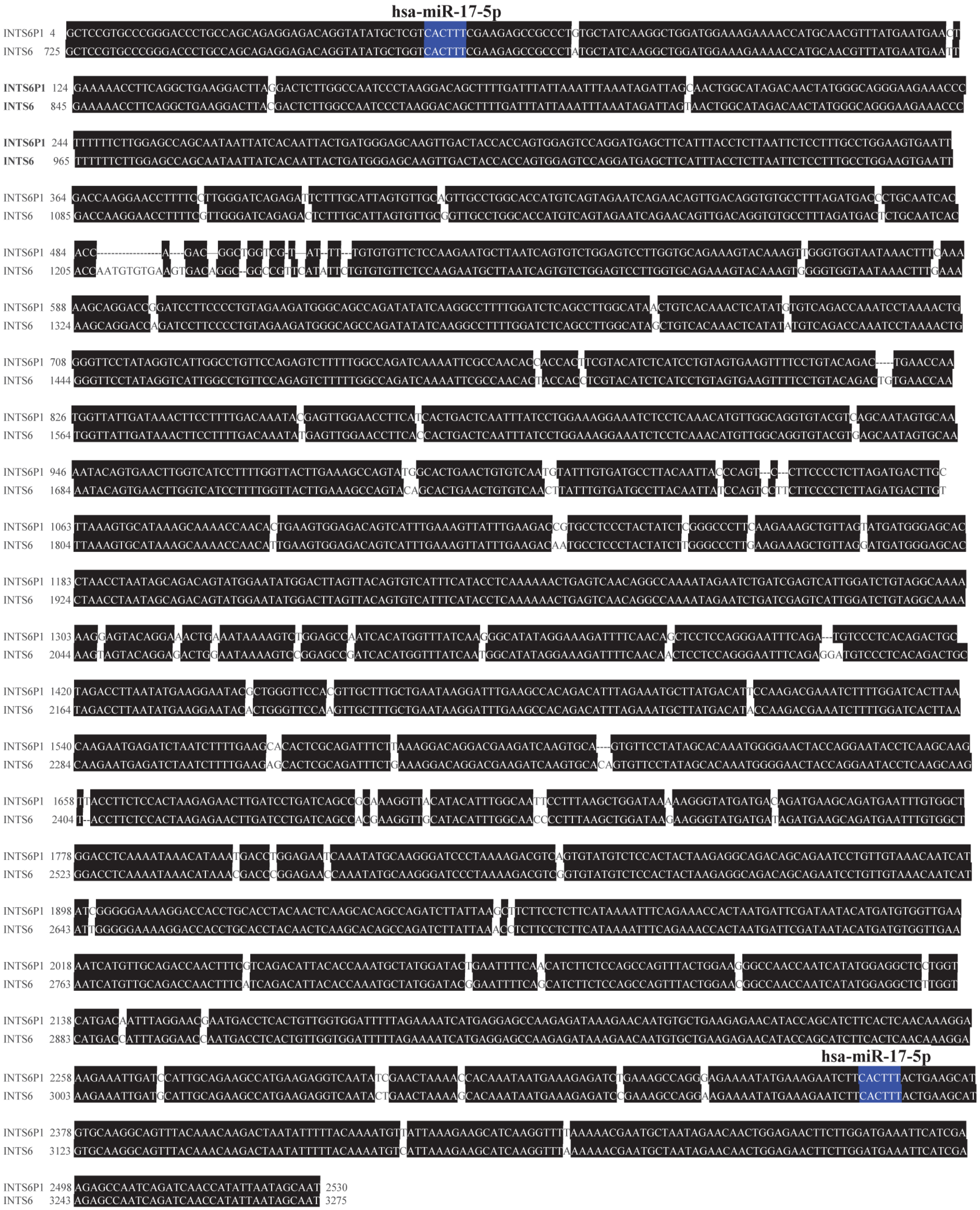

Figure S1. The homology sequence of INTS6P1 and the ORF of INTS6. INTS6P1 shares 96% of the sequence with the ORF of INTS6. Black represents the matched nucleotides between INTS6 and INTS6P1, while the white represents the unmatched. The binding sites of miR-17-5p (blue) are indicated in INTS6 and the ORF of INTS6P1.

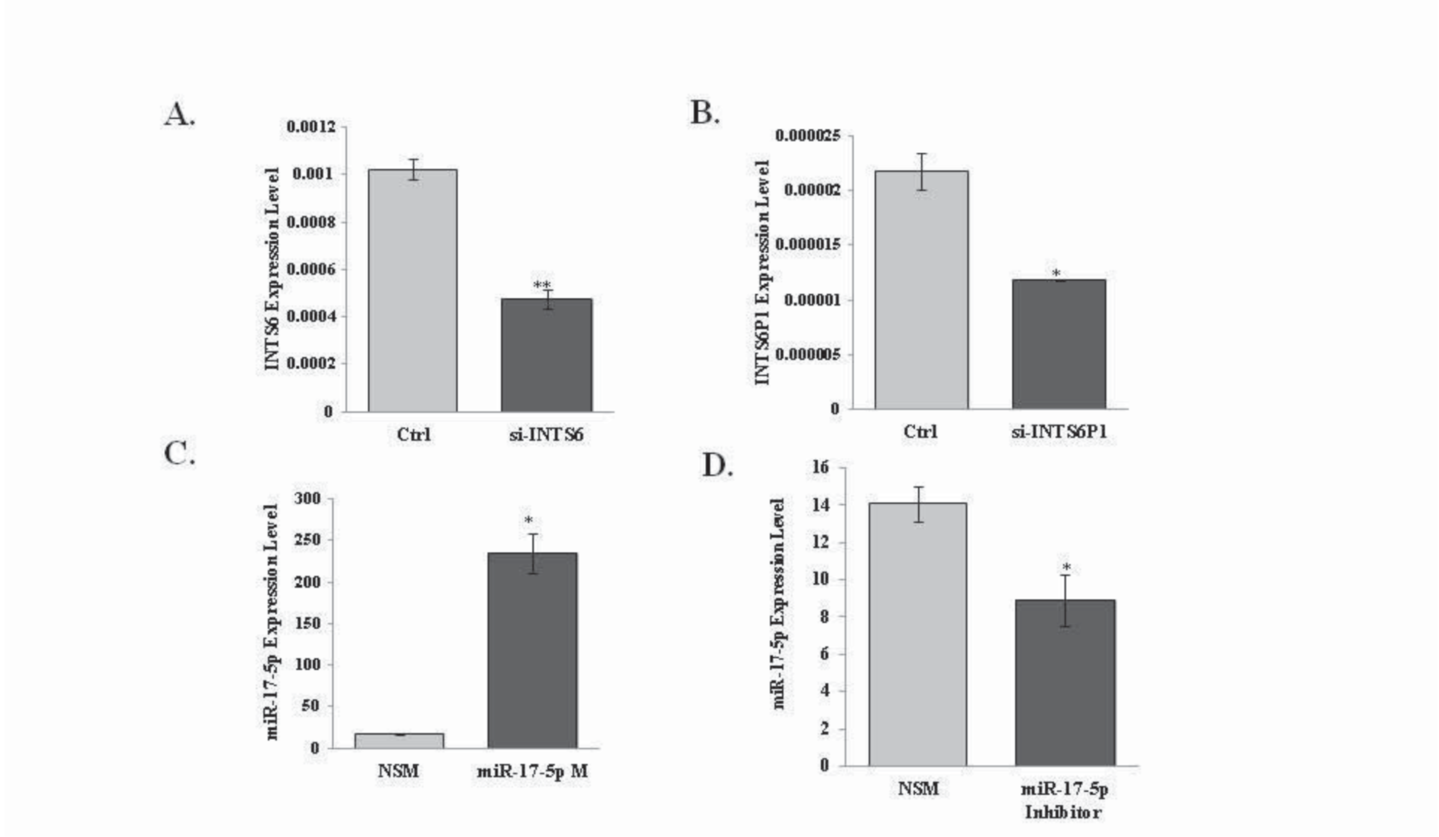

Figure S2. The siRNA, miR-17-5p mimic, and miR-17-5p inhibitor transfection efficiency. (**A**) siRNA-INTS6 (si-INTS6) transfected into MHCC97H cells significantly suppresses the expression of INTS6 *vs.* transfection of siRNA control (Ctrl). (**B**) siRNA-INTS6P1 (si-INTS6P1) transfected into MHCC97H cells significantly suppresses the expression of INTS6P1 *vs.* transfection of siRNA control (Ctrl). (**C**) The expression level of miR-17-5p is increased by transfection of miR-17-5p mimic (miR-17-5p M) *vs.* non specific mimic (NSM). (**D**) The expression level of miR-17-5p is suppressed by transfection with miR-17-5p inhibitor. (**A-D**) The error bars represent mean ± SD.

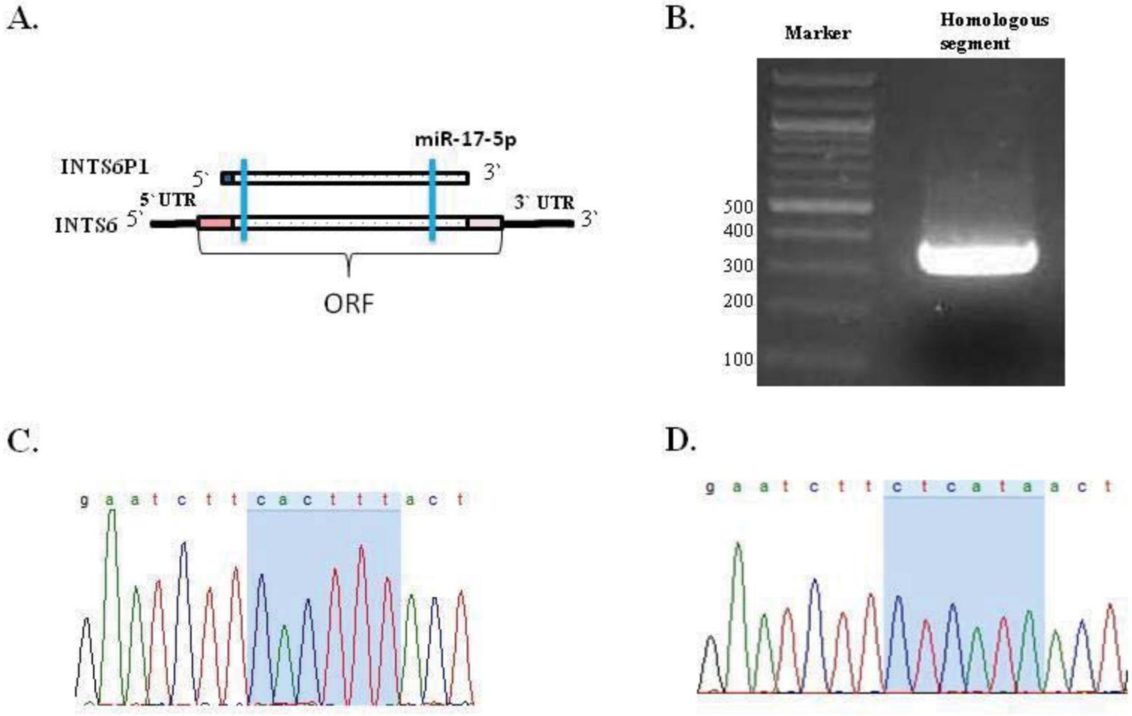

Figure S3. Mutagenesis of miR-17-5p binding site in the homologous sequence of INTS6 and INTS6P1. (**A**) The schematic represents the binding site of miR-17-5p in INTS6P1 and the ORF of INTS6, the 2nd binding site of miR-17-5p in INTS6 and INTS6P1 is coloned to the luciferase vector Pgl4.13. (**B**) The homologous DNA segment (326 bp) is amplified and extracted. This fragment is then cloned into the multiple cloning site of the luciferase vector pGL4.13 XbaI. (**C**) The wild type miR-17-5p binding site (blue highlight) within the 326bp DNA fragment is confirmed by sequencing. (**D**) The mutated binding site for miR-17-5p (blue highlight) is confirmed by sequencing.

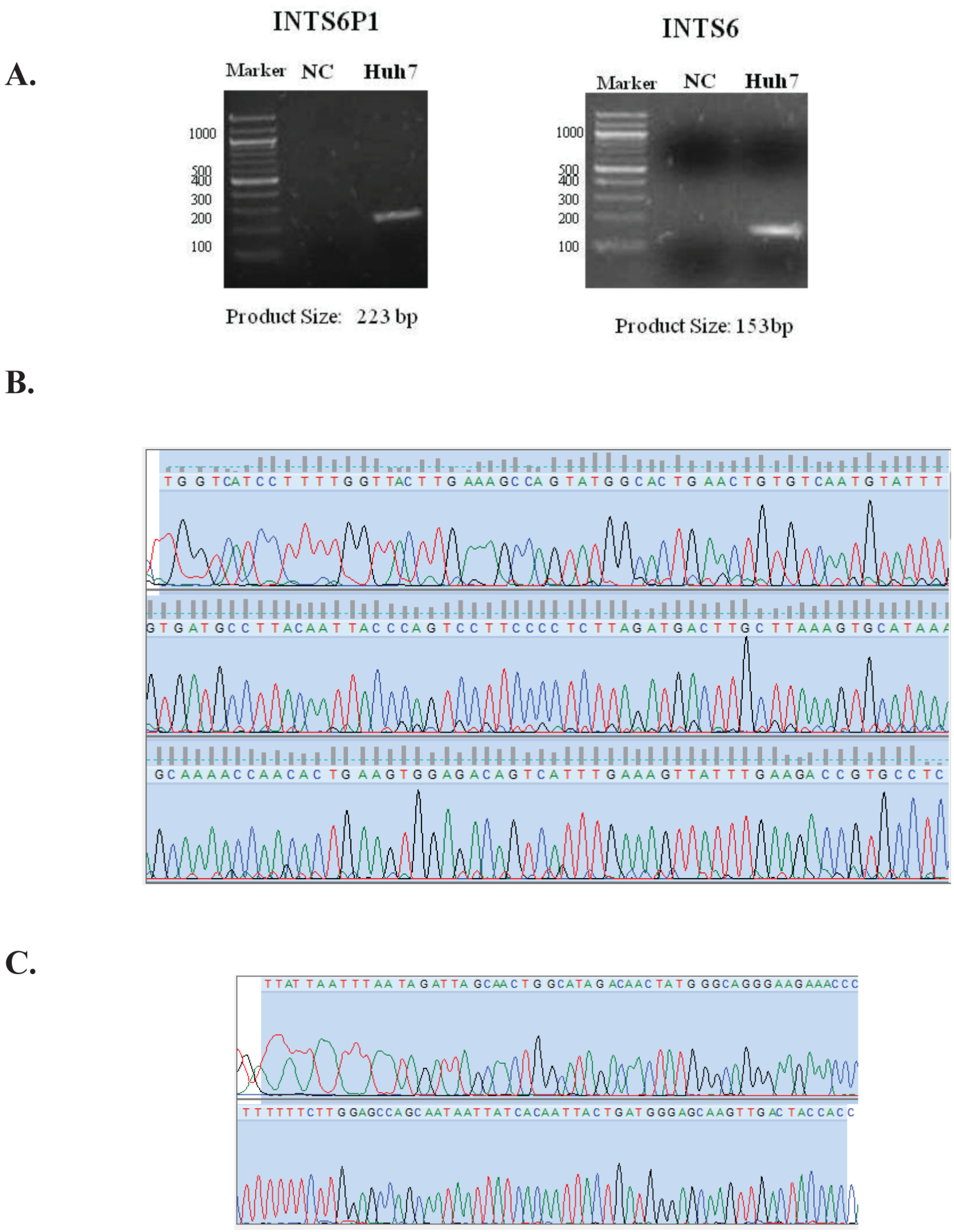

Figure S4. Verifying the qRT-PCR Primers specific. (A) After designing the primers for INTS6P1 and INTS6, PCR was performed to verify the effectivity of the primers. (B) Sequencing the PCR product to verify the specific of INTS6P1 primer. (C) Sequencing the PCR product to verify the specific of INTS6 primer.

Probe Name	Gene Symbol	Fold change	p-value	[1-1, Cancer]	[2-1, Cancer]	[3-1, Cancer]	[1-3, Normal]	[2-3, Normal]	[3-3, Normal]
ASHG19A3A026726	**INTS6P1**	0.335921903	0.014789774	438.8143	165.4434209	183.8416637	768.8101738	717.6663472	859.6026669
NM_001039937	**INTS6**	0.174277385	0.013003468	318.69537	195.11334	179.68677	1428.8856	1064.8241	1485.5529
169336	**hsa-miR-17-5p**	2.709447625	0.016902	299.5	369.6025	244.9138	76.80984	176.7541	83.78015

Table 1. The different expression profile of INTS6P1, INTS6, and miR-17-5p from microarray. The expression level of INTS6P1 and INTS6 is lower in HCC vs. Normal liver tissue. However, the expression level of miR-17-5p is up-regulated in the same HCC vs. Normal liver tissue. Fold change value is calculated by the average value of cancer density divided by the average value of normal liver density.

	Forward	Reverse
**INTS6P1 1ST PCR**	CCTGGTAACCTCGCCTCTGTGCTTC	TCAAATAGCCGACAATGCATGTCAGAA
**INTS6P1 2nd PCR**	ATATGAATTCGAGGAGACAGGTATATGCT	AATTCTCGAGGTTGATCTGATTGGCTCTTC
**INTS6 PCR**	ATATGAATTCGAGGAGACAGGTATATGCT	AATTCTCGAGGTTGATCTGATTGGCTCTTC
**Wild type miR-17-5p binding site**	ATATTCTAGATCATGAGGAGCCAAGAGAT	ATTATCTAGAGTTGATCTGATTGGCTCTTC
**Mutant miR-17-5p binding site**	GAAAGAATCTTCTCATAACTGAAGCATGTGC	GCACATGCTTCAGTTATGAGAAGATTCTTTC
**INTS6**	TTACGACTCTTGGCCAATCC	TGGACTGGACTGTGTTTTTCC
**INTS6P1**	CATGTTGGCAGGTGTACGTC	GGAGGCACGGTCTTCAAATA
**GAPDH**	CATGAGAAGTATGACAACAGCCT	AGTCCTTCCACGATACCAAAGT

Table 2. Primer set sequences utilized for genomic DNA or complementary DNA PCR, mutagenesis, and RNA qRT-PCR .INTS6P1 1st PCR refers to the primers utilized for amplifying INTS6P1 from genomic DNA. INTS6P1 2nd PCR refers to primers utilized for the unique amplification of INTS6P1 (and not INTS6). INTS6 PCR refers to primers utilized to amplify complementary DNA for INTS6. INTS6 and INTS6P1, respectively, refer to primers utilized in RNA qRT-PCR reactions for INTS6 and INTS6P1, respectively.
